# The Cloning and Functional Characterization of Peach *CONSTANS* and *FLOWERING LOCUS T* Homologous Genes *PpCO* and *PpFT*

**DOI:** 10.1371/journal.pone.0124108

**Published:** 2015-04-23

**Authors:** Xiang Zhang, Lijun An, Thi Hung Nguyen, Huike Liang, Rui Wang, Xiayan Liu, Tianhong Li, Yafei Qi, Fei Yu

**Affiliations:** 1 State Key Laboratory of Crop Stress Biology in Arid Areas and College of Life Sciences, Northwest A&F University, Yangling, Shaanxi 712100, People’s Republic of China; 2 Department of Fruit Science, College of Agronomy and Biotechnology, China Agricultural University, Beijing 100193, People’s Republic of China; Wuhan University, CHINA

## Abstract

Flowering is an essential stage of plant growth and development. The successful transition to flowering not only ensures the completion of plant life cycles, it also serves as the basis for the production of economically important seeds and fruits. *CONSTANS* (*CO*) and *FLOWERING LOCUS T* (*FT*) are two genes playing critical roles in flowering time control in Arabidopsis. Through homology-based cloning and rapid-amplifications of cDNA ends (RACE), we obtained full-lengths cDNA sequences of *Prunus persica CO* (*PpCO*) and *Prunus persica FT* (*PpFT*) from peach (*Prunus persica* (L.) Batsch) and investigated their functions in flowering time regulation. *PpCO* and *PpFT* showed high homologies to Arabidopsis *CO* and *FT* at DNA, mRNA and protein levels. We showed that PpCO and PpFT were nucleus-localized and both showed transcriptional activation activities in yeast cells, consistent with their potential roles as transcription activators. Moreover, we established that the over-expression of *PpCO* could restore the late flowering phenotype of the Arabidopsis *co-2* mutant, and the late flowering defect of the Arabidopsis *ft-1* mutant can be rescued by the over-expression of *PpFT*, suggesting functional conservations of *CO* and *FT* genes in peach and Arabidopsis. Our results suggest that *PpCO* and *PpFT* are homologous genes of *CO* and *FT* in peach and they may function in regulating plant flowering time.

## Introduction

The developmental transition from vegetative to reproductive growth is one of the most fundamental events during the life cycles of flowering plants. Given the central role of flowering, it came as no surprise that a large number of genes act in a coordinated manner to ensure the successful induction of the flowering process in the model system *Arabidopsis thaliana* [1–6]. Among the flowering regulatory genes, *CO* *NSTANS* (*CO*) plays a key role in the photoperiod-dependent induction of flowering in Arabidopsis and loss-of-function mutations of *CO* cause a distinctive late flowering phenotype [7–10]. Consistent with its role as a positive regulator of flowering, transgenic plants over-expressing *CO* under the constitutive cauliflower mosaic virus (CaMV) 35S promoter exhibit an early flowering phenotype [11]. *CO* encodes a putative zinc finger transcription factor and CO protein features two conserved domains [7, 12]. The first is a C-X2-C-X16-C-X2-C zinc finger motif in the N terminal region of CO, which is highly homologous to the animal B-box-like transcriptional factors [13, 14]. The other is the so-called CONSTANS, CONSTANS-like and TOC1 (CCT) domain in the C terminus, which may function as a nuclear-localization signal [7, 12]. CO promotes flowering by directly activating the expressions of its downstream genes including *F* *LOWERING LOCUS* *T* (*FT*) and *S* *UPPRESSOR OF* *O* *VEREXPRESSION OF* *C* *O1* (*SOC1*) [15–16]. Similar to *CO*, loss-of-function mutations of *FT* result in delayed flowering, and the over-expression of *FT* can accelerate flowering, indicating that FT is also a flowering activator [17–18]. FT is a small protein of 175 amino acids and a member of the phosphatidyl-ethanolamine binding protein (PEBP) family [17–19]. FT contributes to floral induction by acting as a long distance signal and moves from the leaves to the shoot apex where FT interacts with a bZIP transcription factor, FD, and activate downstream meristem identity target genes such as *LEAFY* (*LFY*) and *APETALA1* (*AP1*) [15, 20–23]. FT protein is believed to represent the so-called “florigen”, which has been quested for ever since the florigen concept was introduced by Chailakhyan in 1937 [24].

The information derived from model plants such as Arabidopsis has been extremely useful to define the molecular mechanisms of how key flowering regulators such as *CO* and *FT* work during flowering induction and has laid the foundation for our current understanding of the mechanisms of flowering control. However, the regulation of flowering in non-model systems such as woody perennial trees remains largely unexplored [3, 25]. Features such as a long juvenile phase and seasonal bud dormancy that are characteristic of many woody plants suggest more complex flowering regulatory schemes [5, 10]. For example, in some species *CO* and *FT* may work as inhibitory signals in non-inductive conditions [26], and they may exert less prominent roles in *Solanum* species [27].

Peach (*Prunus persica* (L.) Batsch) is one of the most economically important fruit producing species and is also emerging as a potential model woody species for genetic studies within the Rosaceae family [28]. In this paper, we report the cloning and functional characterization of peach *CO* and *FT* homologs, *PpCO* and *PpFT*. Gene structure, mRNA and protein sequence analyses showed that *PpCO* and *PpFT* are closely related to Arabidopsis *CO* and *FT*, respectively. We determined that both PpCO and PpFT can be targeted to the nucleus and that PpCO and PpFT have transcription activation activities. Moreover, the ectopic expressions of *PpCO* and *PpFT* can functionally complement the late flowering phenotypes of Arabidopsis *co-2* and *ft-1* mutants, respectively. Our results provide new insight into the functions of *PpCO* and *PpFT* in peach and may enable the future utilization of these genes in the artificial manipulation of peach developmental programs, particularly flowering time regulation.

## Results

### The isolation of *PpCO* and *PpFT* genes

To identify potential expressed peach sequences related to *CO* and *FT* in the public domain, we used *CO* and *FT* homologous sequences from a woody species apple (*Malus domestica*), *MdCO* (NCBI accession number AF052584) and *MdFT* (NCBI accession number AB161112), as the query sequences and searched the NCBI expressed sequence tag (EST) database. We identified one peach EST sequence (NCBI accession number BU044758) homologous to *MdCO* and one peach EST sequence (NCBI accession number BU042239) homologous to *MdFT*. However, these ESTs covered only partial coding sequences of *PpCO* and *PpFT*, respectively.

**Fig 1:**
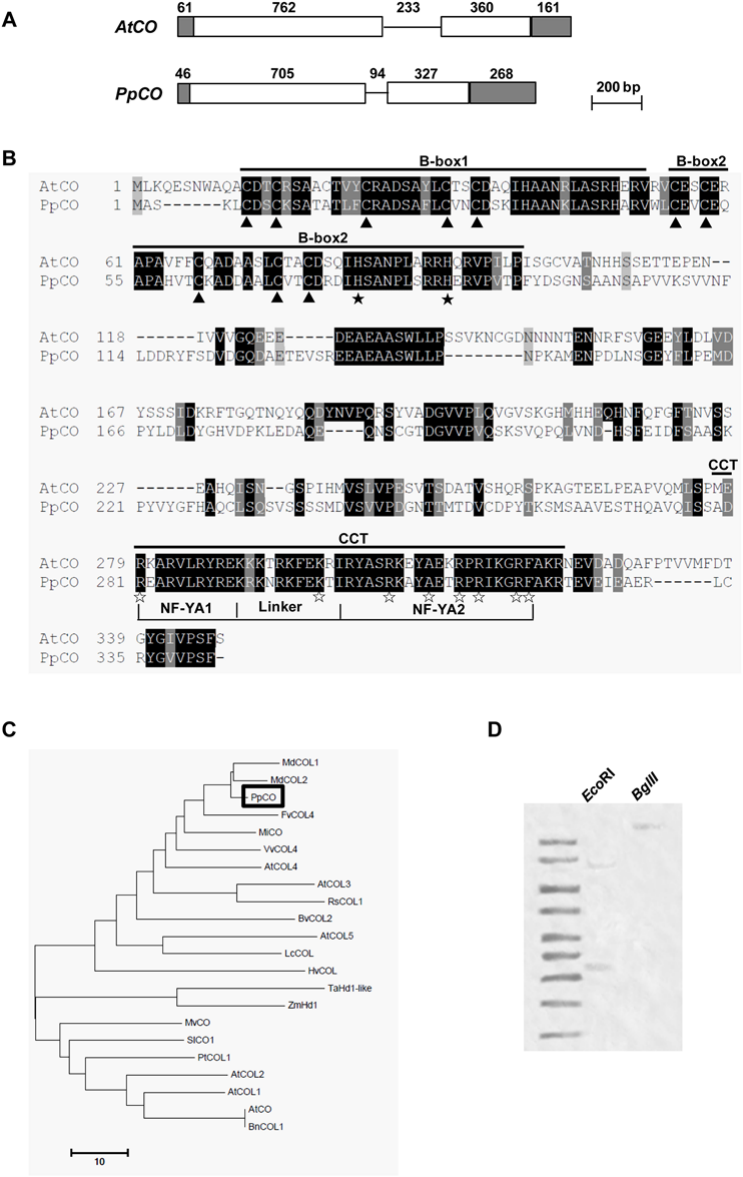
Bioinformatic analysis of peach *PpCO* gene. A. Comparison of gene structures between *PpCO* and *AtCO* from Arabidopsis. The filled boxes indicated the untranslated regions (UTRs), the open boxes indicated the protein coding regions and the lines indicated the introns. Numbers indicated the sizes of UTRs, exons and introns in base pairs. B. Alignment of deduced amino acid sequences of PpCO and AtCO. Amino acids shaded in black indicated highly conserved residues, while those shaded in grey were also conserved. The highly conserved cysteine residues (arrowheads) and histidine residues (filled stars) along with the two consensus B-boxes were marked. The CCT sub-domains NF-YA1, NF-YA2 and their linkers, as well as the conserved residues (open stars) located in these regions, were also indicated. C. Phylogenetic relationship among PpCO and other CO/COL proteins from different species. Bootstrap analysis (1000 replicates) was performed to assess the support of each branch. The accession numbers of the CO proteins used to construct the phylogenetic tree were listed in S2 Table. D. Southern blotting analysis of the *PpCO* gene. Probes used were DIG-labeled full-length *PpCO* cDNAs.

**Fig 2:**
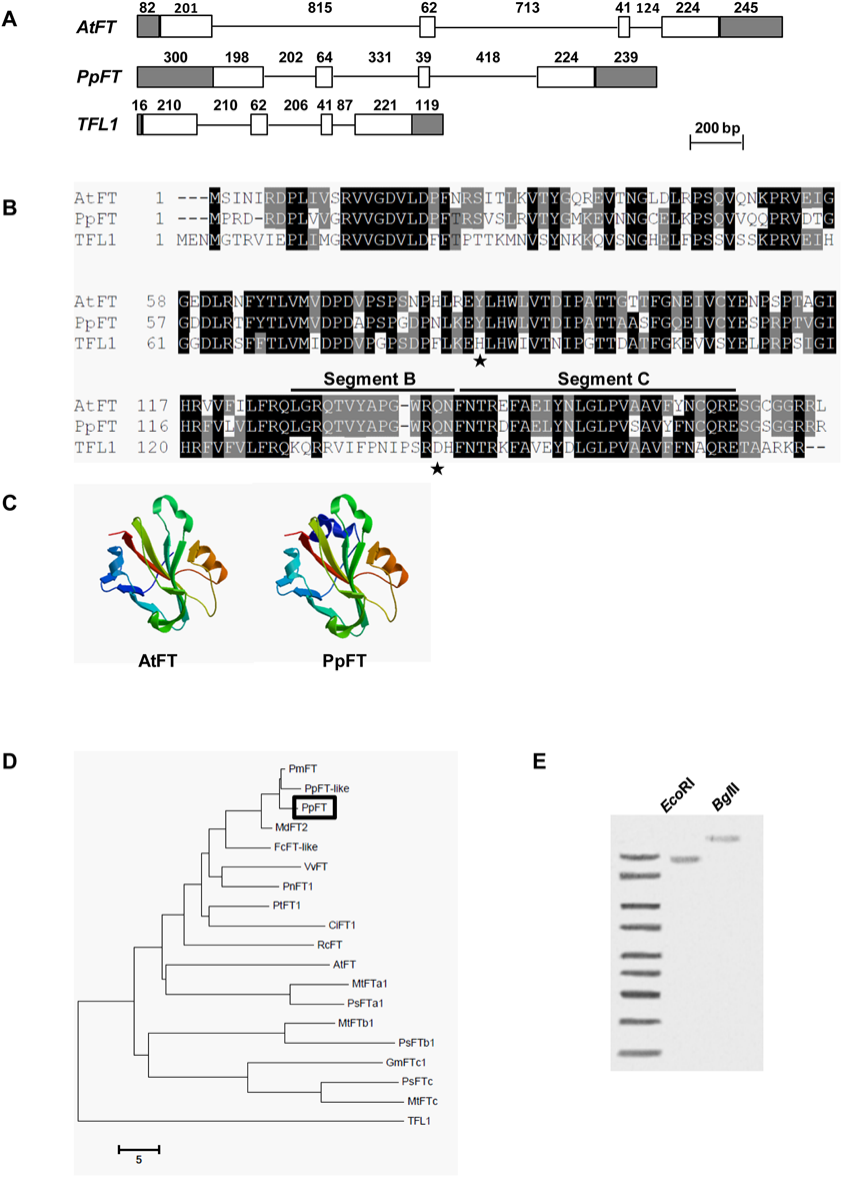
Bioinformatic analysis of peach *PpFT* gene. A. Schematic representation of the genomic organization of *PpFT* from peach, *AtFT* and *TFL1* from Arabidopsis. Gene structures were indicated the same way as those in Fig 1A. B. Alignment of predicted amino acid sequences of PpFT, AtFT and TFL1. The most critical residues for activities (Tyr85 and Gln140 of AtFT, His88 and Asp144 of TFL1) were indicated by filled stars. The functionally important segment B and C encoded by the fourth exon were indicated. C. The predicted 3-D structures of PpFT and AtFT. D. Phylogenetic relationship among PpFT and other FT proteins. Bootstrap analysis (1000 replicates) was performed. The accession numbers of the FT proteins used to construct the phylogenetic tree were listed in S2 Table. PpFT-like is a FT homolog from *Pyrus pyrifolia*. E. Southern blotting analysis of the *PpFT* gene. Probes used were DIG-labeled full-length *PpFT* cDNAs.

To isolate full-length cDNAs of *PpCO* and *PpFT*, we carried out RACE experiments based on the partial *PpCO* and *PpFT* sequences. Using cDNAs synthesized from RNAs extracted from peach mature leaves as templates, we successfully amplified a 353 bp potential *PpCO* fragment and a 188 bp potential *PpFT* fragment that were identical in sequences to BU044758 and BU042239, respectively. Next, based on these two fragments, we obtained the 5′ and 3′ sequences of *PpCO* and *PpFT* using the RACE strategy and assembled open reading frames (ORFs) of the two genes. We next experimentally confirmed the deduced sequences through the direct amplification of full-length cDNAs of *PpCO* and *PpFT* and determined that the ORFs of *PpCO* and *PpFT* were 1032 bp and 525 bp, respectively. Genomic DNA sequences of *PpCO* and *PpFT* were also amplified and *PpCO* and *PpFT* were 1440 bp and 2015 bp in length at the DNA level, respectively (Figs 1A and 2A). The alignment of cDNA sequences and genomic DNA sequences revealed that *PpCO* had a single intron of 94 bp, while *PpFT* had three introns of 202 bp, 331 bp and 418 bp, respectively (Figs 1A and 2A). The full-length cDNA sequences of *PpCO* and *PpFT* were submitted to GenBank under the accession numbers EU939303 and EU939302, respectively.

Based on the cDNA sequences, *PpCO* is annotated to encode a protein of 343 amino acid residues with an estimated molecular weight of ~38 kDa and a theoretical isoelectric point of 6.59. Alignment of the predicted amino acid sequences of PpCO to that of Arabidopsis CO (AtCO) revealed the presence of conserved domains (Fig 1B). PpCO contained two typical C-X2-C-X16-C-X2-C zinc finger structures at the N-terminus, which was highly similar to B-box-type transcription factor such as XNF7 in animals [13–14]. These structures were considered to mediate protein-protein interaction [29]. A conserved CCT domain, which was proposed to serve as a nuclear-localization signal, was also identified in the C-terminus of PpCO protein (Fig 1B) [12]. To dissect the evolution of CO homologs from different plant species, we examined their phylogenetic relationship. PpCO showed between 38.75%-85.17% identities to CO protein sequences from other species, and shared the highest identity with MdCO (85.17%) from apple, which is not surprising as both species belong to the Rosaceae family (Fig 1C).

The deduced protein sequence of PpFT has 174 amino acid residues with a molecular weight of approximately 19.6 kDa and a theoretical isoelectric point of 8.04. PpFT possessed the typical FT protein features including the highly conserved Tyr-84 (PpFT amino acid numbering) in exon 2, Gln-139 (PpFT amino acid numbering), as well as the segment B in exon 4 (Fig 2B). These residues were proposed to lie at the entrance to a putative ligand-binding pocket, and are important for the flowering promoting activity of FT proteins [19]. The predicted 3-D structure of PpFT protein showed a large β sheet in the centre flanked by a small β sheet and an α helix (Fig 2C; http://www.sbg.bio.ic.ac.uk/phyre2). These structural features are similar to those of FT protein from Arabidopsis (AtFT) [19]. Phylogenetic analysis showed that PpFT shared high identities (38.75%-98.28%) with plant FT proteins, especially with PmFT (98.28%) from *Prunus mume* and MdFT (95.04%) from apple (Fig 2D). These data suggest that plant FT proteins are highly conserved, particularly in the Rosaceae family.

The gene copy numbers of *CO* and *FT* members vary among different plant species [10, 30–36]. In order to detect the presence of *PpCO* and *PpFT* genes in peach genome, southern blotting analyses were conducted using full-length *PpCO* and *PpFT* cDNA as probes. Peach genomic DNAs were digested with *Eco*RI and *Bgl*II restriction enzymes, which do not cut either *PpCO* or *PpFT* DNA sequences. Southern blotting results indicated that two *Eco*RI fragments and one *Bgl*II fragment hybridized to the *PpCO* probe (Fig 1D), suggesting at least two copies of *PpCO* were present in the peach genome. One *Eco*RI fragment and one *Bgl*II fragment hybridized to the *PpFT* probe (Fig 2E), indicating that *PpFT* is likely a single copy gene in peach genome.

### PpCO and PpFT show transcriptional activation activity in yeast

**Fig 3:**
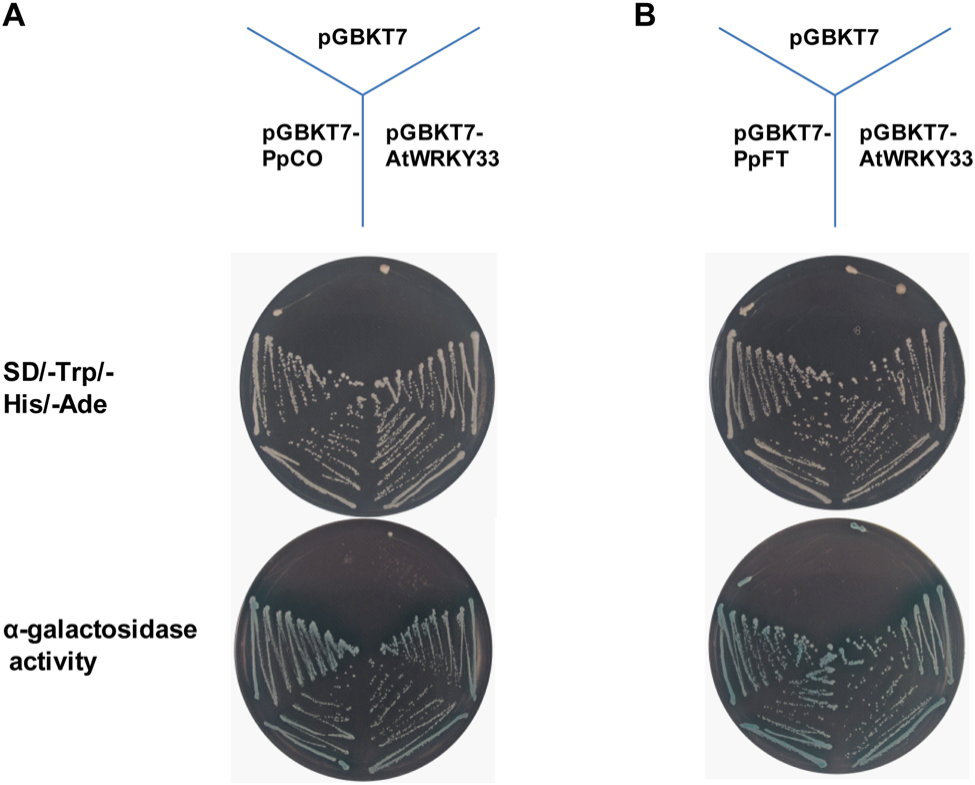
Transcriptional activation analysis of PpCO and PpFT in yeast. PpCO and PpFT were fused with the GAL4 DNA-binding domain and expressed in yeast strain AH109. Transcriptional activation activities were monitored by the detection of yeast growth and α-galactosidase activity. pGBKT7 and pGBKT7-AtWRKY33 were used as negative control and positive control, respectively.

In Arabidopsis, both CO and FT function as transcriptional activators [10, 30] and based on the highly similar gene structures, mRNA and protein sequences, it is likely that PpCO and PpFT may also function as transcriptional activators in peach. We tested potential transcriptional activation activities of PpCO and PpFT through transient expression assays in yeast using a GAL4-responsive reporter system. As shown in Fig 3A and 3B, we observed that the transformed yeast cells harboring pGBKT7-PpCO, pGBKT7-PpFT and pGBKT7-AtWRKY33 (the positive control) grew on the SD medium lacking tryptophan, histidine and adenine whereas cells containing pGBKT7 (the negative control) did not grow, suggesting that PpCO and PpFT can activate the reporter genes (Fig 3A and 3B). We next conducted α-galactosidase activity assays to further validate the growth phenotypes. Consistent with the previous observation, yeast cells transformed with the pGBKT7-PpCO and pGBKT7-PpFT constructs showed clear blue color on plates containing x-α-gal, while pGBKT7 showed negative results (Fig 3A and 3B). These data demonstrate that both PpCO and PpFT could function as transcriptional activators, at least in yeast cells.

### Expression patterns of *PpCO* and *PpFT*

**Fig 4:**
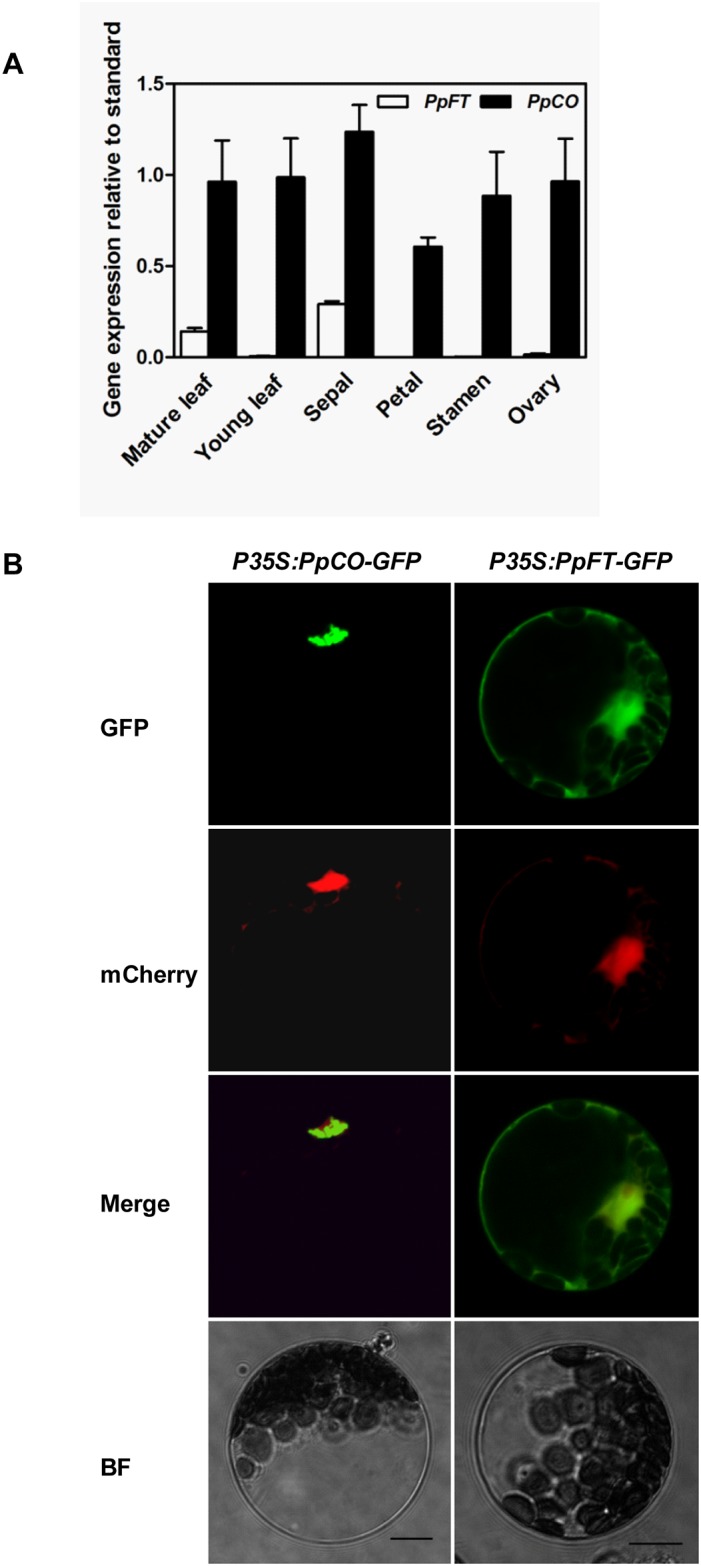
Expression patterns of *PpCO* and *PpFT*. A. Tissue specific expressions of *PpCO* and *PpFT* in peach. Real-time quantitative RT-PCRs were carried out using RNAs from different peach tissues. The data were presented by calculating 2^-ΔCt^. The expressions of *PpACTIN2* were used as controls. B. Subcellular localizations of PpCO and PpFT in Arabidopsis mesophyll protoplasts.

In Arabidopsis, *CO* and *FT* genes function as flowering timing genes, and their transcripts are present mainly in leaves [7, 17]. To investigate the expression patterns of *PpCO* and *PpFT* in peach, we carried out real-time quantitative RT-PCR analysis of the expressions of the two genes in young leaves, mature leaves and different floral organs. As shown in Fig 4A, *PpFT* transcripts were more abundant in mature leaves and sepals, but at markedly lower levels in young leaves and other floral organs. In contrast, *PpCO* transcripts were present in all tissues examined (Fig 4A).

In eukaryotes, many transcriptional activators exert their functions within the nucleus [37]. We thus checked whether this is also the case for PpCO and PpFT through transient expressions of GFP tagged PpCO and PpFT in Arabidopsis leaf protoplasts. Signals of red fluorescent protein mCherry directed by a NLS sequence indicated the nucleus (NLS-mCherry; Fig 4B). For *P35S*:*PpCO-GFP*, GPF signals were observed as a major aggregate in each transformed cell and the signal merged well with NLS-mCherry, suggesting that it is located in the nucleus. For *P35S*:*PpFT-GFP*, GFP signals gave a more diffused distribution (Fig 4B). The majority of PpFT-GFP signals merged with the nucleus but PpFT-GFP signals were also observed in the cytosol (Fig 4B). These data indicate that PpCO and PpFT can localize to the nucleus and consistent with their potential roles as transcriptional activators.

### Functional complementation of *PpCO* and *PpFT* in Arabidopsis

To further validate the roles of *PpCO* and *PpFT* in flowering time regulation, we ectopically expressed *PpCO* under the control of the CaMV 35S promoter in the Arabidopsis *co-2* mutant background, and *PpFT* in the Arabidopsis *ft-1* mutant background, respectively. Independent transgenic lines (>10) were obtained for each transformation, and 5 lines were selected for further analyses. At least 15 plants per line were grown to score the flowering time phenotype. The time of flowering was determined by counting the days to flowering after sowing and the number of rosette leaves at the time of blooming.

**Fig 5:**
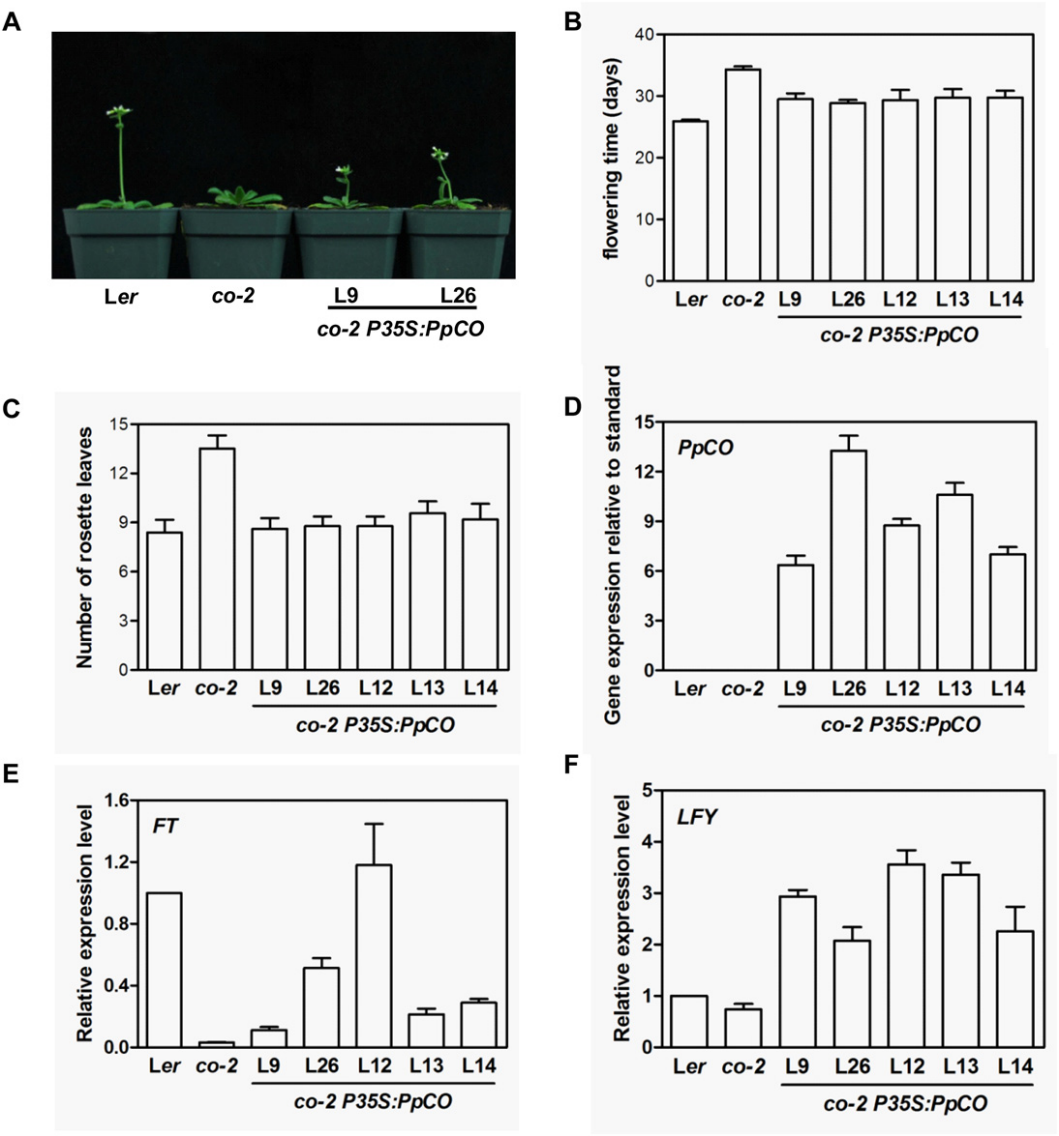
Phenotypes of *co-2 P35S*:*PpCO* transgenic plants. A. Flowering phenotypes of wild type (L*er*), *co* mutant (*co-2*) and *co-2 P35S*:*PpCO* lines. B. Statistics of average days at which the wild type (L*er*), *co* mutant (*co-2*) and transgenic lines started to bloom after sowing. C. Analysis of number of rosette leaves of different genotype plants at blooming. D. Real-time quantitative RT-PCR analysis of transcript accumulations of *PpFT* in different transgenic lines. The data were presented by calculating 2^-ΔCt^. The expressions of *AtACTIN2* were used as controls. E-F. Real-time quantitative RT-PCR analysis of expression levels of *FT* (E) and *LFY* (F) in *co-2 P35S*:*PpCO* transgenic lines. The relative expression levels were first calculated by 2^-ΔCt^, and then divided by their relative expression levels in wild-type, and the ratios were presented. The expressions of *AtACTIN2* were used as controls.

Under our growth conditions, wild type Landsberg *erecta* (L*er*) plants took 25.9±0.2 days to bloom after sowing, while *co-2* mutants (in L*er* background) needed 34.3±0.5 days (Fig 5A, 5B and 5C). In contrast, the time to flowering was reduced to from 28.8±0.5 to 29.8±1.0 days in the five *co-2 P35S*:*PpCO* transgenic lines we examined (Fig 5A and 5B). *PpCO* transcript levels were elevated in transgenic lines (Fig 5D). However, we did not observe a correlation between the *PpCO* transcript level and the degree of flowering time rescue. Our results indicate that the over-expression of *PpCO* can, at least partially, compensate for the lack of Arabidopsis *CO* in *co-2* mutants.

**Fig 6:**
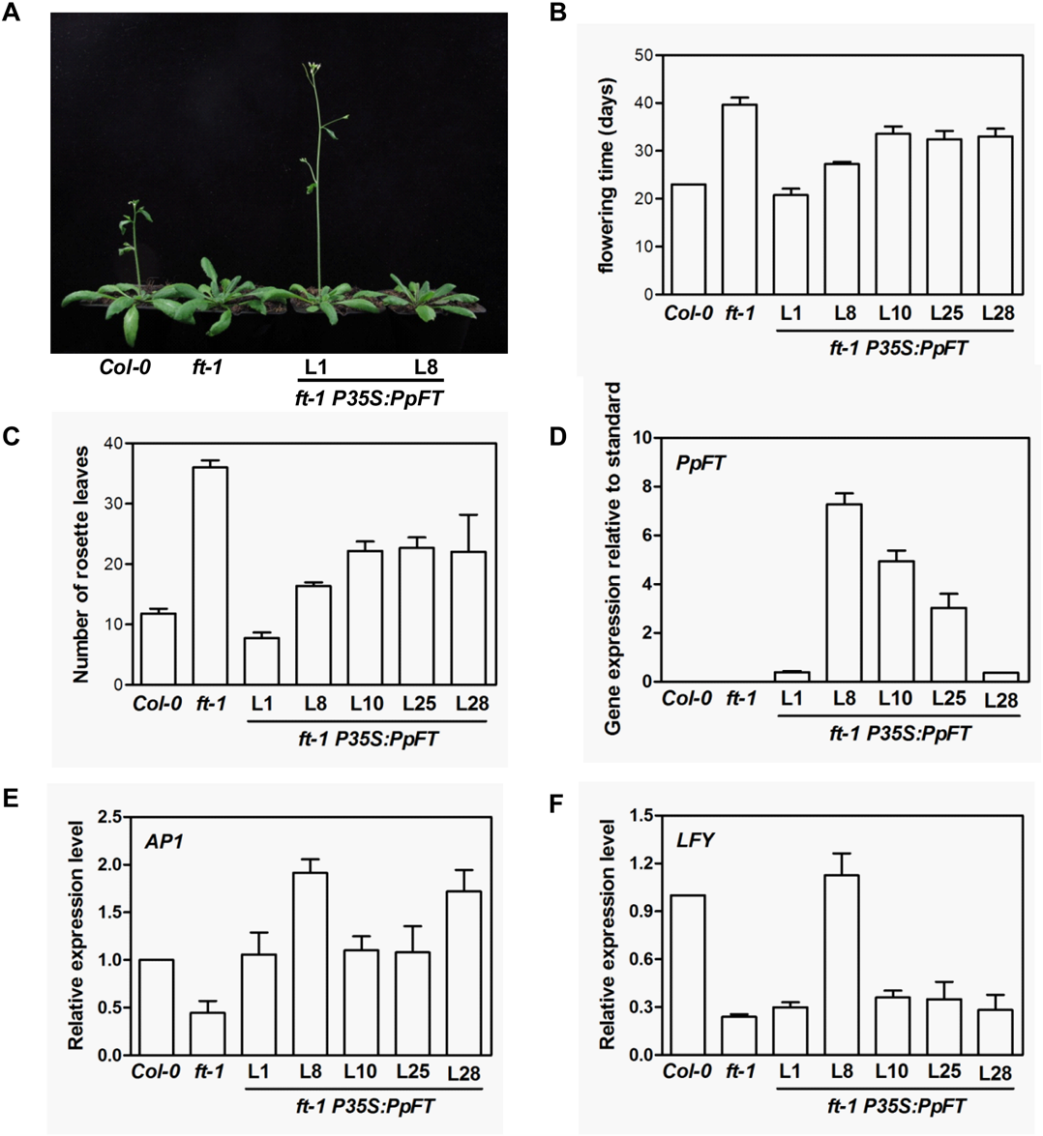
Phenotypes of *ft-1 P35S*:*PpFT* transgenic plants. A. Flowering phenotype of wild type (Col-0), *ft* mutant (*ft-1*) and *ft-1 P35S*:*PpFT* lines. B. Statistics of average days at which the wild type (Col-0), *ft* mutant (*ft-1*) and transgenic lines started to bloom after sowing. C. Analysis of numbers of rosette leaves of different genotype plants at blooming. D. Real-time quantitative RT-PCR analysis of transcript accumulations of *PpFT* in different transgenic lines. The data were presented by calculating 2^-ΔCt^. The expressions of *AtACTIN2* were used as controls. E-F. Real-time quantitative RT-PCR analysis of expression levels of *AP1* (E) and *LFY* (F) in *ft-1 P35S*:*PpFT* transgenic lines. Calculation of the relative expression levels was the same as that in Fig 6E and 6F. The expressions of *AtACTIN2* were used as controls.

For the Arabidopsis *ft-1* mutant, which is in the Columbia (Col-0) background, it took 39.6±1.4 days to flower, whereas the wild type Col-0 plants flowered at 23.0±0.0 days (Fig 6A, 6B and 6C). On the contrary, *P35S*:*PpFT* lines we examined took from 20.7±1.3 to 33.5±1.5 days to enter flowering (Fig 6A, 6B and 6C). As expected, the expression levels of *PpFT* in different transgenic lines were significantly increased (Fig 6D). However, the expression levels of *PpFT* and the degree of *ft-1* flowering time rescue were not very correlated, similar to what we observed in *co-2 P35S*:*PpCO* lines. These data suggested that *PpFT* probably shares similar molecular functions with *FT* in Arabidopsis.

To further dissect the downstream events of *PpCO* and *PpFT* over-expressions, we examined the expressions of *CO* downstream genes *FT* and *LFY*, and *FT* downstream gene *AP1*. In *co-2* mutants, *FT* and *LFY* transcript levels were lower than those in wild type (Fig 5E and 5F). As shown in Fig 5E and 5F, in *co-2 P35S*:*PpCO* lines, the transcript levels of *FT* (Fig 5E) and *LFY* (Fig 5F) were clearly increased compared to those in *co-2* mutants. In *ft-1* mutants, *AP1* expression was reduced compared to that of wild type (Fig 6E). In contrast, the expression levels of *AP1* were increased in the *ft-1 P35S*:*PpFT* lines (Fig 6E). The expression levels of *LFY* were not significantly changed except for line 8 (Fig 6F). These results suggest that the expression of *PpCO* and *PpFT* can functionally complement the corresponding Arabidopsis mutants and activate downstream gene expressions, and they may function as positive flowering regulators in peach.

## Discussion

The control of flowering is a central process in higher plants and is of enormous importance in the breeding efforts of economically important perennial fruit trees [3–4, 6, 28]. Among cultivated woody fruit trees, peach is an emerging model species for isolating and characterizing genes for key agronomical traits because of its relatively small genome size [26, 28]. Identifying flowering regulatory genes and establishing their genetic interactions in peach will help to paint a comprehensive picture of the flowering process in woody species. In addition, the artificial application of these genes in peach will be beneficial to both breeders for faster productions of hybrids and shorter breeding cycles, and farmers in terms of better cultivar adaption and stable annual productions.

In this study, through EST database mining and subsequent cloning of full-length cDNA sequences, we isolated and characterized a *CO* homolog *PpCO* and a *FT* homolog *PpFT* from peach. Several lines of evidence suggest that PpCO and PpFT may function as transcription factors and promote flowering in peach.

Firstly, we established that *PpCO* and *PpFT* share high homologies with Arabidopsis *CO* and *FT* at many levels. At the genomic DNA level, *PpCO* shared a similar gene structure with Arabidopsis *CO* (Fig 1A). At the protein level, PpCO was evolutionary conserved with CO-like proteins from many plant species (Fig 1B and 1C). Features such as the B-box containing the conserved C-X2-C-X16-C-X12-C motif at the N-terminus, the critical Cys and His residues, the consensus spacing, and the CCT domain in the C-terminus were also present in the PpCO protein [10]. In Arabidopsis, there are 17 members in the CO family, which can be further catalogued into three subgroups [12]. Group I members contain two B boxes, group II has only one B box, and group III members have a conserved B box and a variant of B box. These B-boxes are related to domains present in transcription factors from animals and other organisms and are likely involved in protein-protein interactions [29]. Based on its N-terminus protein sequence, PpCO likely belongs to group I and is closely related to CO in Arabidopsis (Fig 1C).

A similar case of homology was also observed between *PpFT* and Arabidopsis *FT*. The gene structure of *PpFT* resembled that of Arabidopsis *FT* (Fig 2A). PpFT protein is also highly conserved with FT proteins from Arabidopsis and other plant species (Fig 2B and 2D). Moreover, the predicted 3-D structure of PpFT is also very similar to that of FT with a large central β-sheet flanked on one side by a smaller β-sheet and on the other by an α-helix (Fig 2C). Protein sequence variations within the FT-like family members could dramatically affect their ability to promote or repress flowering [19, 30, 33]. In Arabidopsis, Tyr-85 and Gln-140 (FT numbering) as well as residues within the segment B are indispensable for its activity to promote flowering. TERMINAL FLOWER 1 (TFL1), a FT-like protein, the amino acid residues equivalent to Tyr-85 and Gln-140 (FT numbering) are changed to His-88 and Asp-143, respectively, which leads to its function of a flowering repressor protein [19, 38]. Similar findings were also demonstrated with BvFT1 and BvFT2 in sugar beet [39]. PpFT shared the most of conserved amino acid residues with Arabidopsis FT, including the critically important Tyr-84 (equivalent to Tyr-85 in FT), Gln-139 (equivalent to Gln-140 in FT) and the conserved residues equivalent to those in segment B of FT protein, i.e., Tyr-133/Tyr-134, Gly-136/Gly-137, Trp-137/Trp-138 (PpFT numbering/FT numbering; Fig 2B). The highly conserved residues like Asp-73, Pro-75, Tyr-85, His-87, Gly-109, His-118, Val-120, Arg-139, which contribute to the anion-binding site in FT are also present in PpFT (Fig 2B and 2C) [19]. These data are consistent with the notion that both FT and PpFT may function as activators of flowering. Overall, the conserved nature of PpCO and PpFT suggests that they may be functionally related with CO and FT in Arabidopsis.

Secondly, we tested whether PpCO and PpFT fit the criteria for potential transcription factors since they share high protein homologies with their Arabidopsis counterparts CO and FT, which both act as transcription factors [7, 12, 15, 20–21]. Transient expression analyses in Arabidopsis protoplasts demonstrated that PpCO and the majority of PpFT protein were localized to the nucleus, consistent with their potential roles as transcription factors (Fig 4B) [7–8]. In addition, transcriptional activation activity assays showed that both PpCO and PpFT demonstrated transcriptional activation activities in yeast cells (Fig 3). Taking together, these data suggest that PpCO and PpFT may function as transcription factors in peach.

Lastly, we characterized the functions of *PpCO* and *PpFT* in the model system *Arabidopsis thaliana*. In Arabidopsis, CO and FT integrate flower induction signals and promote the transition to flowering, and mutants of *CO* and *FT* show a late flowering phenotype [5, 30]. To investigate the functions of *PpCO* and *PpFT* in planta, we expressed these genes in corresponding Arabidopsis mutants and generated *co-2 P35S*:*PpCO* and *ft-1 P35S*:*PpFT* transgenic plants, respectively. We found that ectopic expressions of *PpCO* and *PpFT* could effectively rescue the late flowering phenotype of *co-2* and *ft-1* mutants, respectively, indicating that *PpCO* and *PpFT* can functionally complement Arabidopsis *CO* and *FT* (Figs 5 and 6). These findings are consistent with reports for transgenic Arabidopsis or tobacco plants containing *CO* and *FT* or their homologs from other plant species [10, 30, 35]. In Arabidopsis, *CO* can activate the expressions of *FT* and *LFY* [9], while *FT* could induce the expression of *AP1* but not *LFY* in promoting flowering [15]. We found that the expression levels of *FT* and *LFY* were elevated in *co-2 P35S*:*PpCO* plants (Fig 5E and 5F). In addition, *AP1* transcripts were increased in *ft-1 P35S*:*PpFT* plants while *LFY* expressions were not changed in most *ft-1 P35S*:*PpFT* lines (Fig 6E and 6F). Our results are consistent with findings in Arabidopsis and suggest that PpCO and PpFT can activate downstream target genes of Arabidopsis CO and FT. In addition, *co-2 P35S*:*PpCO* and *ft-1 P35S*:*PpFT* transgenic plants had normal vegetative growth and flower morphologies, and this is in line with previous reports of transgenic Arabidopsis plants containing CO and FT or their homologs from other plant species [3, 5, 30]. Taken together, our results suggest that ectopic expressions of *PpCO* and *PpFT* can promote flowering in Arabidopsis.

In summary, we identified full-length cDNA sequences of *PpCO* and *PpFT* from a woody species peach. Sequence analyses showed that PpCO and PpFT share conserved domains with CO and FT members from Arabidopsis and other plant species. Functional characterizations revealed that PpCO and PpFT likely locate to the nucleus and have transcriptional activation activities. In addition, ectopic expressions of *PpCO* and *PpFT* can rescue the late flowering phenotypes of Arabidopsis *co-2* and *ft-1* mutants, defective in *CO* and *FT*, respectively. Thus *PpCO* and *PpFT* likely represent the functional counterparts of *CO* and *FT* in peach and may participate in the regulation of flowering.

## Materials and Methods

### Plant Materials

Peach variety *Prunus persica* (L.) Batsch. cv. Bayuecui was used in this study. The peach trees were grown in the Institute of Forestry and Pomology, Beijing Academy of Agricultural and Forestry Science, P. R. China (latitude: 39°97′45.43″N; longitude: 116°22′66.58″E). Because it is one of the research partners of China Agricultural University, there is no specific permission required for research sampling in this institute. In addition, the peach cultivar we used is not an endangered or protected species. Peach leaves were harvested on May 30, 2012, frozen in liquid nitrogen immediately and stored at -80°C until use. Leaves at the tip of the shoot were designated as young leaves while those at the base of the shoot were designated as mature leaves.

Wild type *Arabidopsis thaliana* ecotypes Columbia-0 (Col-0) and Landsberg *erecta* (L*er*) were used in this study. Arabidopsis flowering mutants *co-2* (L*er* background) and *ft-1* (Col-0 background) have been described [40]. Arabidopsis plants were cultured under continuous illumination (~100 μmol m^-2^ s^-1^) in a controlled growth room maintained at ~22°C.

### DNA and RNA Procedures

Total RNAs were extracted from the mature leaves using the Trizol RNA reagent (Life technologies, USA). CTAB method was used for total genomic DNA purification. Southern blot analysis using digoxin (DIG)-labeled probes was carried out according to manufacturer’s manual (Mylab Corporation, China).

First strand cDNAs were synthesized using the PrimeScript reverse transcription kit (Takara, Japan) according to the manufacturer’s instructions. *PpCO* and *PpFT* gene specific primers PpCOF1, PpCOR1, PpFTF1 and PpFTR1 were designed according to the peach *CO* and *FT* EST sequences (Accession number: BU044758 for *PpCO*, BU042239 for *PpFT*) deposited in the National Center for Biotechnology Information (NCBI) and used to amplify partial cDNAs of *PpCO* and *PpFT*. Full-length cDNAs of *PpCO* and *PpFT* were then obtained via 5′ and 3′ RACE methods using a Generacer kit (Life Technologies, USA) following the manufacturer’s manual. Primers PpCOF2, PpCOF3, PpCOR2, PpCOR3 were used to obtain *PpCO* full-length cDNAs and primers PpFTF2, PpFTF3, PpFTR2, PpFTR3 were used to obtain *PpFT* full-length cDNAs. Amplified fragments were cloned to pMD18-T vector (Takara, Japan) and sequenced in both directions. Detailed information of primers used in this study was listed in S1 Table.

For Real-time quantitative RT-PCR analysis, total RNAs were extracted using Trizol RNA reagent (Life Technologies, USA) and 1 μg DNase I treated RNA was used for cDNA synthesis. Real-time PCRs were performed with a Bio-Rad CFX96 Real-Time PCR System. Relative expression levels of the target genes were calculated with 2^-Δct^. Primers used (PpCOF7 and PpCOR7 for *PpCO*, PpFTF7 and PpFTR7 for *PpFT*, FTF and FTR for *AtFT*, LFYF and LFYR for *LFY*, AP1F and AP1R for *AP1*) in real-time PCR experiments were listed in S1 Table. Expressions of *PpACTIN2* and *AtACTIN2* genes were used as controls.

### Bioinformatics Analysis

Open reading frames (ORFs) and protein sequences of *PpCO* and *PpFT* were predicted with the NCBI ORF Finder program. The isoelectric point and molecular weight of PpCO and PpFT protein were calculated using the PeptideMass program (http://web.expasy.org/peptide_mass/). Multiple protein sequence alignments were carried out using Clustal x 1.83 and GeneDoc software (http://iubio.bio.indiana.edu/soft/molbio/ibmpc/genedoc-readme.html). Phylogenetic trees were constructed by the MEGA5 program using the Neighboring-Joining method (http://www.megasoftware.net/).

### Yeast Transcriptional Activation Assays

The ORFs of *PpCO* and *PpFT* were obtained by PCR with gene specific primers (PpCOF4 and PpCOR4 for *PpCO*; PpFTF4 and PpFTR4 for *PpFT*) and cloned into the *Eco*RI and *Pst*I sites of BD vector pGBKT7 to generate pGBKT7-PpCO and pGBKT7-PpFT. Sequences of primers used were showed in S1 Table. Each of the BD vectors including pGBKT7-PpCO, pGBKT7-PpFT, negative control vector (pGBKT7) and positive control vector (pGBKT7-AtWRKY33) [41] was used to transform yeast strain AH109. Activities of *MEL-1* reporter genes were assayed following the procedures described in Yeast Protocols Handbook (Clontech, USA).

### Arabidopsis Protoplast Transient Expression Assays

The ORFs of *PpCO* and *PpFT* were obtained by PCR with gene specific primers (PpCOF5and PpCOR5 for *PpCO*; PpFTF5 and PpFTR5 for *PpFT*) and cloned into the *Sal*I site of the pTF486 vector [42] to generate *P35S*:*PpCO-GFP* and *P35S*:*PpFT-GFP* for protoplast transient expression. Detailed primer information was described in S1 Table. Arabidopsis leaf protoplast isolation and transformation were performed as described in [42]. Cellular localizations of GFP-tagged proteins were examined with confocal microscope (Nikon, Japan).

### Functional Complementation of the Arabidopsis *co* and *ft* Mutants

Full-lengths *PpCO* and *PpFT* cDNAs were amplified using gene specific primers (PpCOF6 and PpCOR6 for *PpCO*; PpFTF6 and PpFTR6 for *PpFT*) and cloned into *Nco*I and *Bgl*II sites of pCAMBIA1301 binary vector (CAMBIA, Australia) to generate *P35S*:*PpCO* and *P35S*:*PpFT*. Primers sequences was showed in S1 Table. *P35S*:*PpCO* and *P35S*:*PpFT* were introduced into the Arabidopsis *co-2* and *ft-1* mutant background, respectively. The floral dip method was used for Arabidopsis transformation [43]. Transgenic plants were screened on half strength Murashige and Skoog agar plates supplemented with 50 mg L^–1^ hygromycin. Phenotypes of the transgenic lines were examined in T2 generation.

## Supporting Information

S1 Table Primers used semi-quantitative PCR analysis. (DOCX)

S2 Table Accession numbers of the deduced amino acid sequences used in Figs 1 and 2. (DOCX)
