# Supplementary material for: The Cloning and Functional Characterization of Peach CONSTANS and FLOWERING LOCUS T Homologous Genes PpCO and PpFT
Source: PLoS One. 2015 Apr 23;10(4):e0124108. doi: 10.1371/journal.pone.0124108 (PMC4408105; doi:10.1371/journal.pone.0124108)
Supplement: S1 Table — (DOCX) [file pone.0124108.s001.docx]

**Supporting information**

**Table S1 Primers used semi-quantitative PCR analysis**

| Name | Sequences |
| --- | --- |
| PpCOF1: | 5´-AGCTTGAAGATGCTCAGGAGCA-3´ |
| PpCOR1 | 5´-TCGGTTCTTTCGCTTCTCTCTG-3´ |
| PpFTF1 | 5´-AACGGTGGGGATTCATCGCTTT-3´ |
| PpFTR1 | 5´-ATCTTCTCCTCCCTCCAGAGCC-3´ |
| PpCOF2 | 5´-AGCTTGAAGATGCTCAGGAGCA-3´ |
| PpCOF3 | 5´-AACACGACGATGACGGACGTGTG-3´ |
| PpCOR2 | 5´-TCGGTTCTTTCGCTTCTCTCTG-3´ |
| PpCOR3 | 5´-ACCGCAACTGTTCTGCTCCTGA-3´ |
| PpFTF2 | 5´-AACGGTCCCCATTCATCGCTTT-3´ |
| PpFTF3 | 5´-GCGGCTCTGGAGGGAGGAGA-3´ |
| PpFTR2 | 5´ATCTTCTCCTCCCTCCAGAGCC-3´ |
| PpFTR3 | 5´-GCATACACTGTTTGCCTACCCA-3´ |
| PpCOF4 | 5´-CGGAATTCATGGCGTCGAAGCTCTGT-3´ |
| PpCOR4 | 5´-ATGACGTCACTAGAACGACGGAAC-3´ |
| PpFTF4 | 5´-CGGAATTCATGCCTAGGGACAGGGAC-3´ |
| PpFTR4 | 5´-ATGACGTCATTATCTTCTCCTCCCTCCA-3´ |
| PpCOF5 | 5´-CGGTCGACATGGCGTCGA-3´ |
| PpCOR5 | 5´-TAGTCGACACCACCACCACCACCACCGAACGACGGAACGACGCC-3´ |
| PpFTF5 | 5´-CGGTCGACATGCCTAGGGACAGGGAC-3´ |
| PpFTR5 | 5´- TAGTCGACACCACCACCACCACCACCTCTTCT CCTCCCTCCAGA-3´ |
| PpCOF6  PpCOR6  PpFTF6  PpFTR6  PpCOF7  PpCOR7  PpFTF7  PpFTR7  FTF  FTR  LFYF  LFYR  AP1F  AP1R  AtACTIN2F  AtACTIN2R  PpACTIN2F  PpACTIN2R | 5´-CCATGGAAATGCCTAGGGACAGGGA-3´  5´-AGATCTGATCTTCTCCTCCCTCCA-3´  5´-CCATGGAAATGGCGTCGAAGCTCTGTG-3´  5´-AGATCTGAGAACGACGGAACGACGCCG-3´  5´- GGTTCAGAGCAAAAGCGTTC -3´  5´- GCGCTCATTGACTTGGTGTA -3´  5´- CCGCTTGTTGTTGGAAGAGT -3´  5´- AGGATCCACCATGACCAGAG -3´  5´-CTGGAACAACCTTTGGCAAT -3´  5´-AGCCACTCTCCCTCTGACAA -3´  5´-ATTGGTTCAAGCACCACCTC -3´  5´-CAAGAAGCTCCCAACGAAAG -3´  5´-GCAAGCAATGAGCCCTAAAG -3´  5´-ACTGCTCCTGTTGAGCCCTA -3´  5´- CTTGCACCAAGCAGCATGAA -3´  5´- CCGATCCAGACACTGTACTTCCTT -3´  5´-CTGCCATGTATGTTGCCATC-3´  5´-GACGAAGAATGGCATGAGGT -3´ |
