# Supplementary material for: The Cloning and Functional Characterization of Peach CONSTANS and FLOWERING LOCUS T Homologous Genes PpCO and PpFT
Source: PLoS One. 2015 Apr 23;10(4):e0124108. doi: 10.1371/journal.pone.0124108 (PMC4408105; doi:10.1371/journal.pone.0124108)
Supplement: S2 Table — (DOCX) [file pone.0124108.s002.docx]

**Table S2 Accession numbers of the deduced amino acid sequences used in figure 1 and figure 2.**

| Name | Accession number | |
| --- | --- | --- |
| *CO* | | NP_197088´ |
| COL1  COL2  *COL3*  *COL4*  *COL5*  *BnCOL1*  *Hv* *COL*  *Rv COL1*  *Tv Hd1-like*  *Zm Hd1*  *PtCOL1*  *MdCOL2*  *SlCO1*  *MvCO*  *FvCOL4*  *VvCOL4*  *MdCOL1*  *LcCOL*  *BvCOL2*  *MiCO*  *PmFT*  *MdFT2*  *Fc FT-like*  *VvFT*  *PnFT1*  *PtFT1*  *RcFT*  *MtFTa1*  *PsFTa1*  *PsFTc*  *MtFTc*  *GmFTc1*  *MtFTb1*  *PsFTb1*  *CiFT1*  *TFL1*  *AtFT*  *PpFT-like* | | NP_197089  NP_186887  Q9SK5  Q940T9.2  Q9FHH8  AAN09813  AAM74069  AAC35496  BAC92733  ABW82153  AGM20690  XP_008341828  NP_001233839  ACV88633  XP_004291989  XP_002263458  NP_001280817  AGS32266  ACC95130  ACN62415  AM943979  AB458504  BA160052  DQ871590  AB10611  XM_002334269  CBY25182  XP_003624569  ADZ05699  ADZ05703  XP_003624575  NP_001239994  AE199553  ADZ05701  AB027456  NP_196004  NM_105222  AB524587 |
